# Safety for Miners after Explosions

**Published:** 1906-10-20

**Authors:** 


### Safety for Miners after Explosions.

The appalling explosion which has caused the death of several miners at the Wingate Colliery in Durham is a reminder of the dangers which attend the workers in mines. It is not generally realised how large a proportion of the men killed in a colliery explosion die from suffocation caused by the irrespirable gases or "damps" which are found in mines. A large number of miners lose their lives every year solely for the want of a temporary supply of oxygen. Choke damp is another name for black-damp, though it is sometimes used to designate after-damp. Black-damp or choke-damp differs from air in the fact that it contains a slightly greater percentage of carbonic acid and a slightly less percentage of oxygen, the diminution of oxygen being more marked than the increased carbonic acid. The danger from choke-damp arises from a deficiency of oxygen. There may be little or no warning of its action on the body before the limbs and senses are paralysed. The lamp which the miners carry fortunately announces the danger, and should prevent them from walking into an atmosphere of choke-damp. When the quantity of oxygen is diminished to about 3 per cent. the light goes out. A diminution of 11 per cent. of oxygen is required before life is endangered. The so-called "white-damp" may have fatal effects on a miner, and yet not extinguish his lamp. Mr. Dickinson before the 1879 Commission described after-damp as "having a pungent gaseous taste and an irritating action on the eyes, producing a feeling of malaise and torpor, which is a warning of imminent danger. Those who get too far into it lose the power over their legs, fall down, and are suffocated through lack of oxygen while in this helpless condition." This appears to have been the case with the miners killed in the Wingate explosion.

Death causes the appearance of suffocation and occurs as follows: When the carbonic acid present in the air reaches 3 per cent. there is an increase in the number of respirations, which gradually extend until 18 per cent. is reached, and the distress becomes acute. These indications are independent of the quantity of oxygen present, provided there is not less than 10 per cent. The action of the increased carbonic acid would be to prevent entirely the free interchange of gases in the lungs, so that the blood would become intensely charged with the products of combustion. The distress caused by diminishing the oxygen below 10 per cent. is less acute than in the case of increased carbonic acid. The latter constitutes a serious danger, for the danger point, as in the case of various anæsthetics, is reached quite suddenly. The effects are not felt by the person subjected to the action of these gases. All at once the loss of motive power occurs, followed by paralysis of the cerebellar function and loss of consciousness. Eight or nine respirations of air containing but 2 per cent. of oxygen will render a person insensible without any previous inconvenience having been felt.

Authorities are agreed that the loss of life in explosions in coal-mines is due to the want of oxygen, and if imprisoned miners could be supplied with sufficient oxygen to last them for twenty minutes they would be enabled after an explosion to pass through the fire-damp by the various passage-ways which lead to the bottom of the shaft and obtain the requisite supply of oxygen from the atmosphere. It does not seem a difficult feat to provide the sixty or seventy litres of oxygen required for an hour's respiration to each worker in a mine. The Davy lamp is supplied to him as a protection. Dr. Haldane contends that at an expenditure of 6d. a miner could be supplied with a small steel tube containing the requisite amount of oxygen to last for an hour, and an india-rubber connection with a regulating valve would enable it to be brought into operation when its use was required. It is useless to urge that the mining men are so little careful of their own safety that they would object to carrying such a life-saving agent into the seams and galleries in which they have to work. The safety appliance might be deposited close to where the men were working in small pockets or pouches near their pit clothes, so that they could always be at hand when required. The whole subject has been carefully gone into by Dr. J. S. Haldane, but we regret to say that his recommendations on the subject have so far been generally ignored by proprietors and managers of the mines and by those who are generally responsible for the safe conduct of the mining industry.

